# Fragment-Based Screen of SARS-CoV-2 Papain-like Protease (PL^pro^)

**DOI:** 10.1021/acsmedchemlett.4c00238

**Published:** 2024-07-23

**Authors:** Ashley J. Taylor, Kangsa Amporndanai, Tyson A. Rietz, Bin Zhao, Anusha Thiruvaipati, Qiangqiang Wei, Taylor M. South, Mackenzie M. Crow, Chideraa Apakama, John L. Sensintaffar, Jason Phan, Taekyu Lee, Stephen W. Fesik

**Affiliations:** 1 Department of Biochemistry, Vanderbilt University School of Medicine, Nashville, Tennessee 37232-0146, United States; 2 Department of Pharmacology, Vanderbilt University School of Medicine, Nashville, Tennessee 37232-6600, United States; 3 Department of Chemistry, Vanderbilt University, Nashville, Tennessee 37235, United States

**Keywords:** SARS-CoV-2, PL^pro^, antiviral, fragment-based drug discovery, NMR screening

## Abstract

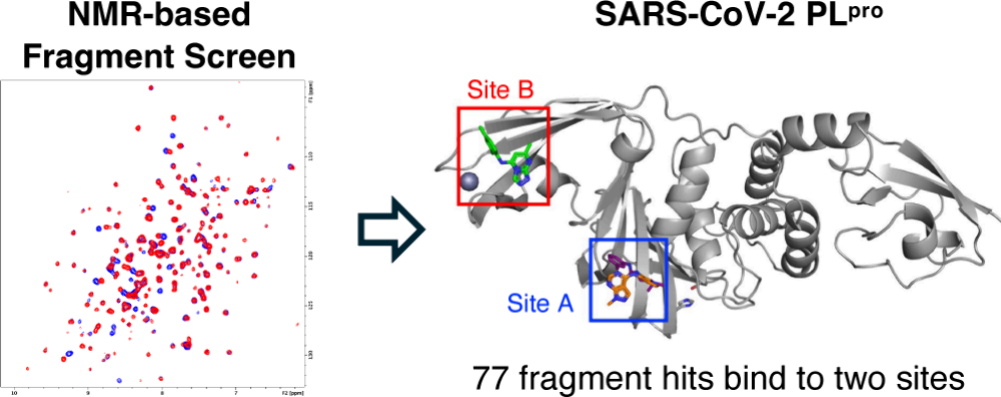

Coronaviruses have been responsible for numerous viral outbreaks in the past two decades due to the high transmission rate of this family of viruses. The deadliest outbreak is the recent Covid-19 pandemic, which resulted in over 7 million deaths worldwide. SARS-CoV-2 papain-like protease (PL^Pro^) plays a key role in both viral replication and host immune suppression and is highly conserved across the coronavirus family, making it an ideal drug target. Herein we describe a fragment-based screen against PL^Pro^ using protein-observed NMR experiments, identifying 77 hit fragments. Analyses of NMR perturbation patterns and X-ray cocrystallized structures reveal fragments bind to two distinct regions of the protein. Importantly none of the fragments identified belong to the same chemical class as the few reported inhibitors, allowing for the discovery of a novel class of PL^Pro^ inhibitors.

The Covid-19 pandemic caused by SARS-CoV-2, the present and future variants of this virus, and the potential for other coronaviruses to cause outbreaks highlight the need for antiviral drugs targeting critical proteins in the coronavirus life cycle. Currently, there are three FDA approved drugs for the treatment of Covid-19: two viral RNA dependent RNA polymerase inhibitors (Remdesivir and Molnupiravir)^1,2^ and one viral main protease (M^pro^) inhibitor (Nirmatrelvir).^3^ Although these drugs have drawbacks/limitations affecting their ability to be a widely useful treatment for SARS-CoV-2 infections, other polymerase and main protease inhibitors are under active development. As expected, SARS-CoV-2 mutants have developed resistance against Remdesivir and Nirmatrelvir in cellular passaging assays and in drug treated Covid-19 patients.^4−6^ This suggests that additional antiviral treatments are needed against new viral targets that act through different mechanisms of action.

The SARS-CoV-2 genome is a single stranded RNA of ∼30 000 nucleotides which encodes for 4 structural (spike, membrane, envelope, and nucleocapsid proteins) and 16 nonstructural proteins (NSP1-16).^7,8^ Following host infection, SARS-CoV-2 translates its genome in two open reading frames into two polyproteins, which require subsequent cleavage by cysteine proteases to generate functionally active nonstructural proteins. The main protease and papain-like protease (PL^Pro^) are responsible for the cleavage of nonstructural proteins 4–16 and 1–3, respectively. Both enzymes are considered essential for viral replication and maturation.^9,10^ Although inhibitors of the main protease have been developed, no inhibitors of PL^Pro^ have reached the clinic. The high homology of PL^Pro^ across the coronavirus family makes PL^Pro^ an attractive drug target to overcome drug-resistant variants or new emerging coronaviruses in future.^11^

In addition to viral replication PL^Pro^ plays a role in host immune evasion through the cleavage of the ubiquitin and interferon-stimulated gene 15 (ISG-15).^12^ ISG-15 is a 15 kDa protein comprised of two ubiquitin-like domains that is strongly induced by type 1 interferons and bacterial and viral infections.^13−15^ While the exact mechanism for ISG-15 inhibition of viral infections is unknown, ISGylation of viral proteins has been found to effect replication, maturation, and egress across various viral species.^16^ Additionally, in vivo studies where ISG-15 expression has been suppressed have shown higher rates of viral growth and increased mortality.^17,18^ PL^Pro^ cleaves the C terminus of ISG-15 (RLRGG) with sub-μM affinity allowing for the virus to delay the immune response,^19^ resulting in increased levels of infectivity for SARS-CoV-2 compared to other members of the coronavirus family.^12^

Previous efforts to target SARS-CoV-1 have led to the discovery of GRL-0617 which also weakly inhibits SARS-CoV-2.^20,21^ Recently, analogues of GRL-0617 have been reported by several research groups which show improved inhibition against PL^Pro^ (Figure 1).^22−25^ However, despite numerous drug discovery campaigns, no novel chemical scaffolds have been identified for SARS-CoV-2 inhibitors. The substrate recognition sequence of PL^Pro^ (LXGG)^26^ poses a significant challenge to the discovery and development of covalent PL^Pro^ inhibitors due to the S1 and S2 subsites forming a narrow tunnel blocking access to the catalytic triad.

**Figure 1:**
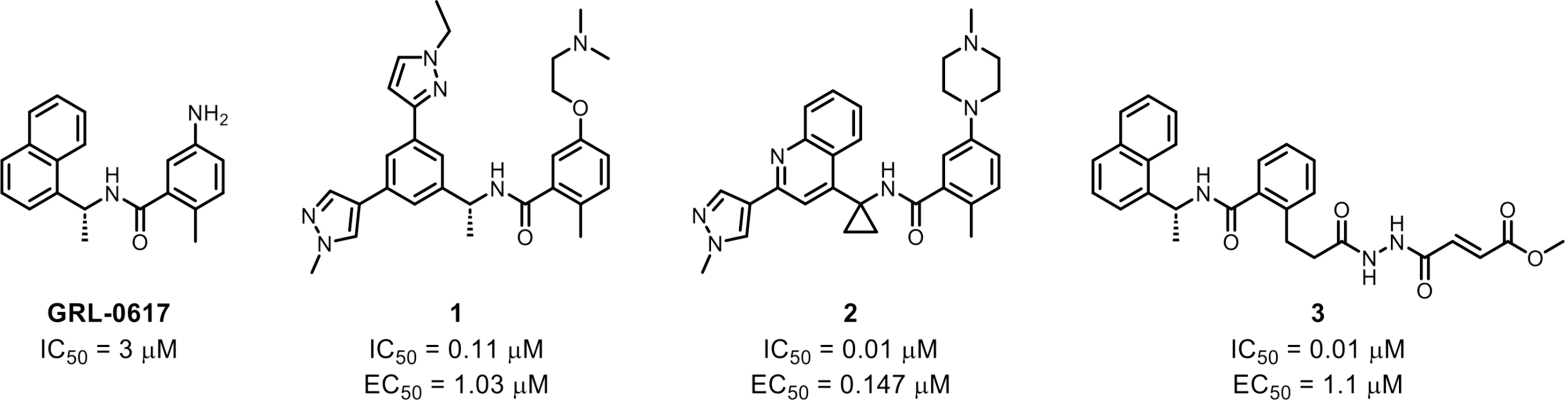
Structure of the first reported PL^Pro^ inhibitor GRL-0617^21^ and its analogues developed by Rutgers University^22^**1**, Pfizer^24^**2**, and Oak Ridge National Laboratory^25^**3** with their inhibitory and cellular activity reported.

In this paper, we describe a fragment-based screen of a truncated papain-like protease from SARS-CoV-2 using protein-observed NMR. This fragment-based screening method to discover new PL^Pro^ ligands is advantageous due to its ability to detect weak binding fragments, measure binding affinity without a secondary assay, and differentiate binding based on chemical shift patterns. In this screen, we have identified several unique hits that bind to the active site and additional molecules that bind to a site in another subdomain, as shown by X-ray crystallography.

In order to obtain a high-quality NMR spectrum of PL^Pro^, the protein was truncated to remove the ubiquitin-like (Ubl) domain (residues 1–70), reducing protein size while maintaining the binding affinity at the active site. In addition, this construct (residues 71–314) also contained two mutations (C111S and C270S) to improve stability and reduce aggregation at high concentrations. An in-house 13,824 molecule fragment library was screened against uniformly ^15^N-labeled recombinant SARS-CoV-2 PL^Pro^ using protein-observed ^1^H/^15^N SOFAST-HMQC NMR.^27^ Fragments were initially screened as mixtures containing 12 fragments, with a concentration of 0.8 mM per fragment. All spectra were manually inspected for chemical shift perturbations (CSP). The hits of fragment mixtures were identified if there were visual changes in chemical shifts for the backbone resonances caused by fragments compared with the reference spectrum of ligand-free PL^Pro^. Individual fragments from 12-compound mixture hits were rescreened as singletons to identify the actual fragments that bind to PL^Pro^. A total of 77 fragment hits that bind to SARS-CoV-2 PL^Pro^ were discovered in this screen, with a hit rate of 0.56%. Based on the literature, a protein with greater than a 0.1% fragment hit rate is suggested to be a druggable target for small molecules.^28^ Two distinct chemical shift patterns were observed for the fragments (Figure 2A,B). This suggests that our fragment hits bind to two distinct pockets on PL^Pro^. We classify these two groups of hits as class A or class B according to each shift pattern. To rank the potency of the hits, a SOFAST-HMQC titration was used to calculate binding affinities (*K*_d_) by measuring the CSP in the presence of 0.0625–2 mM fragment hits. Example CSP and titration curves are given in Figure S1. Twenty-two hits showed a *K*_d_ of less than 1 mM. Representative hits that bind to site A are shown in Figure 2C, and those that bind to site B are shown in Figure 2D.

**Figure 2:**
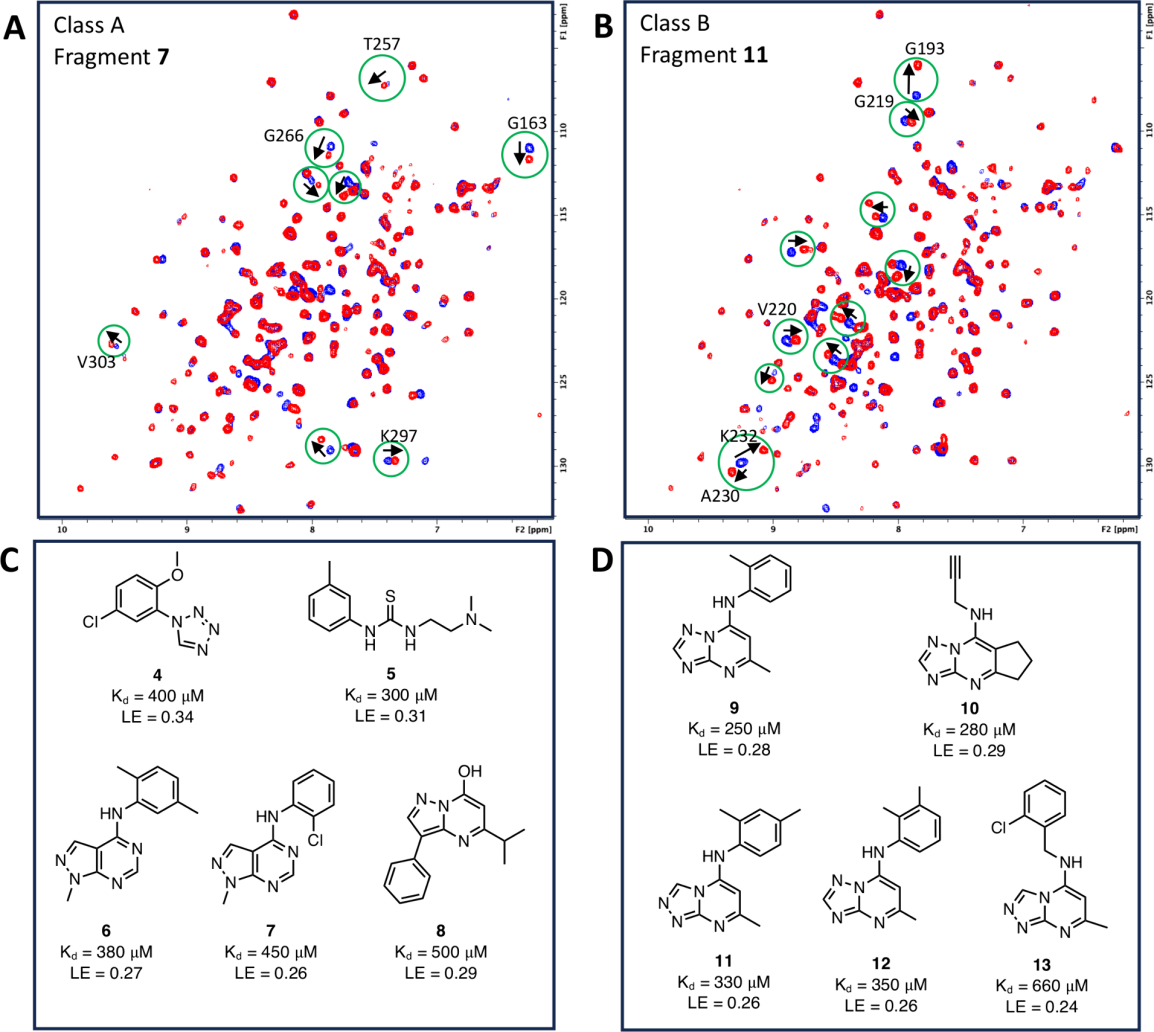
Fragment hits identified in the NMR-based fragment screen. ^1^H–^15^N SOFAST HMQC spectra of PL^Pro^ without (blue) and with (red) 0.8 mM fragment hits illustrate the different chemical shift changes caused by (A) the class A and (B) class B hits. Characteristic peak shifts of each class of fragment were highlighted in green circles. Labeled peak assignments are given in panels A and B sourced from literature.^29^ Representative structures of the (C) class A and (D) class B fragment hits with their binding affinities (K_d_) measured by NMR titration and calculated ligand efficiency (LE).

To determine the binding site of the two fragment classes, GRL-0617, a PL^pro^ competitive inhibitor, was tested against PL^Pro^ and was found to give a chemical shift pattern indicative of the class A fragments, suggesting that they are binding to the active site. This is further supported by the fact that when 1 mM of a class A fragment was added to a sample that was previously incubated with 0.06 mM GRL-0617, no changes in the NMR spectrum were observed (Figure 3A), suggesting that GRL-0617 can outcompete the binding of class A fragments. However, when 1 mM of a class B fragment was added to a sample incubated with 0.06 mM GRL-0617, extra chemical shifts indicative of a class B binder were observed (Figure 3B). This confirms that the class B fragments are binding to a distinct region of the protein different from the class A fragments and that binding is not mutually exclusive.

**Figure 3:**
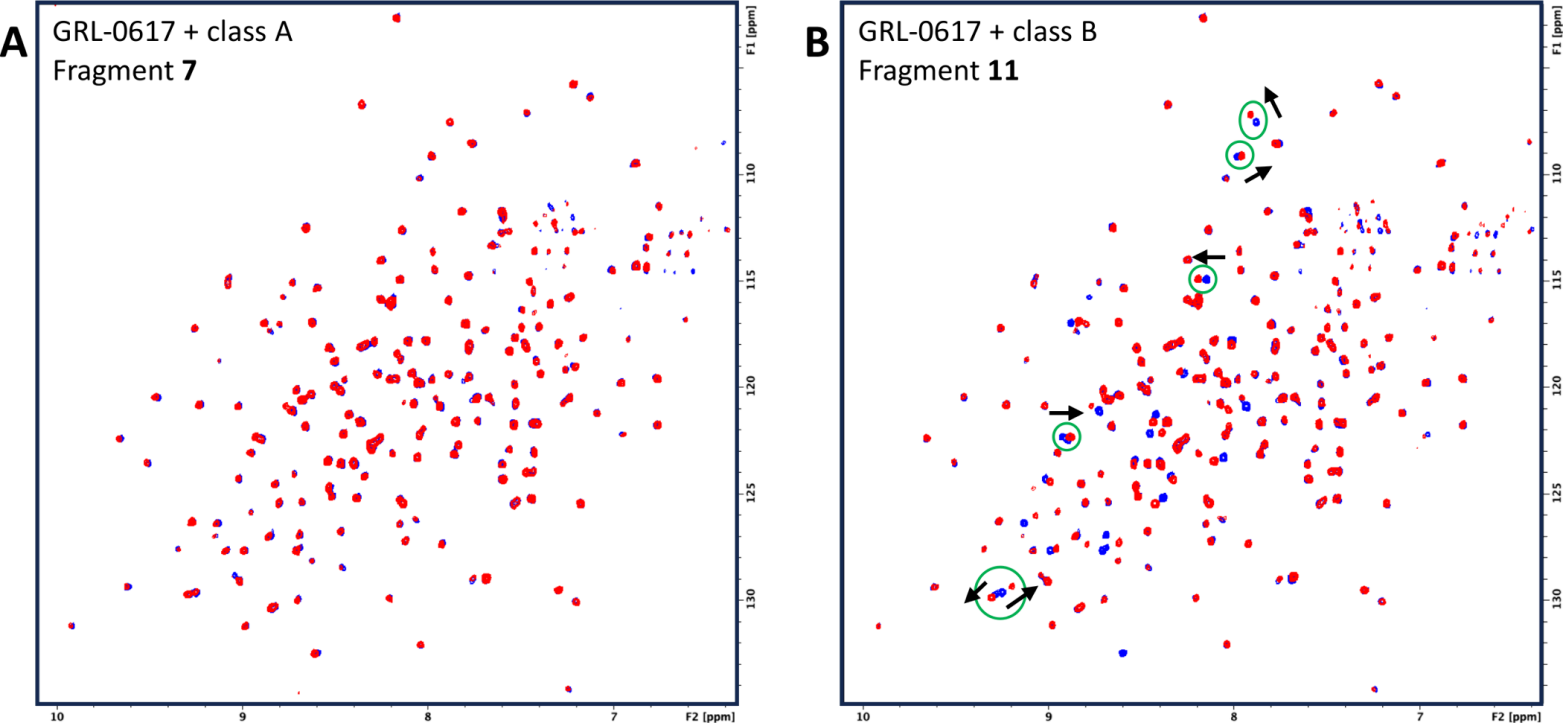
^1^H–^15^N SOFAST HMQC spectra of PL^Pro^ incubated with 0.06 mM GRL-0617 (blue) and 0.06 mM GRL-0617 + 1 mM fragment (red) from (A) a class A fragment hit **7** and (B) a class B fragment hit **11**.

X-ray crystallography was utilized to further clarify the binding mode of our fragment hits and aid in the design of fragment analogues. Although we were not able to obtain crystal structures with the NMR protein construct, the full-length protein (residues 1–314) containing two cysteine to serine mutations (C111S and C270S) did produce suitable crystals for X-ray diffraction with structures for 3 different protein-fragment complexes being solved. The X-ray data collection and structure refinement statistics are in Table S1, and the electron density maps of fragments are exhibited in Figure S2. The X-ray structures confirmed our NMR studies, showing class A fragments bound at the S4 subsite adjacent to the BL2 loop region, while class B compounds bound at a previously undocumented binding site in the finger region (Figure 4A). Based on the B-factor analysis of the apo PL^Pro^ structure (PDB ID: 7D47), we observed that the pocket for the class A compounds is more rigid area than the class B pocket (Figure 4B).

**Figure 4:**
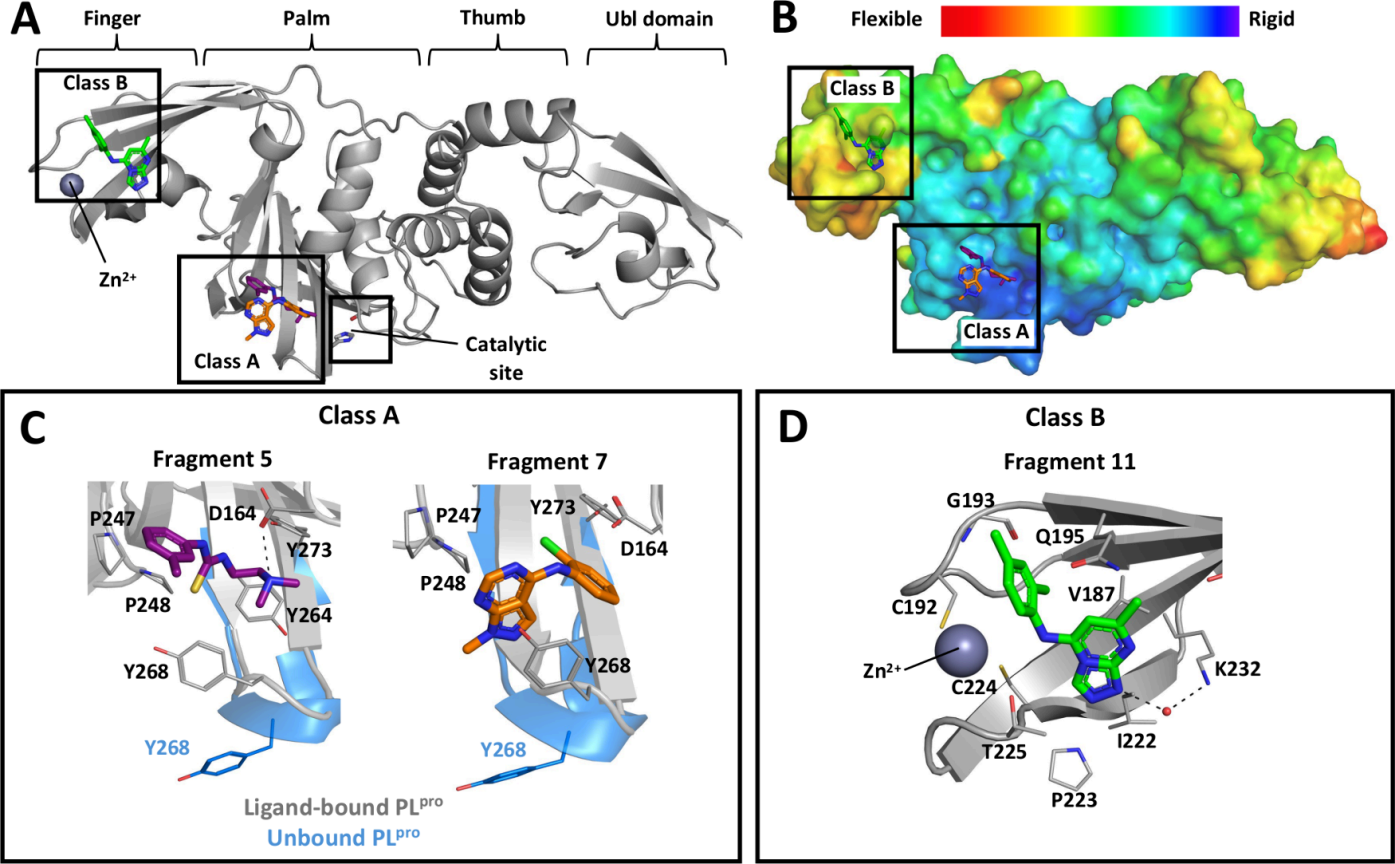
X-ray crystal structures of SARS-CoV-2 PL^Pro^ with fragments. (A) Class A and class B fragments bound to PL^Pro^ at palm and finger domains, respectively. (B) B-factor map of apo PL^Pro^ structure (PDB ID: 7D47). Binding pockets in (C) palm domain occupied by class A fragments (PDB ID: 9BRV and 9BRW for **5** and **7**, respectively) and (D) finger domain occupied by class B fragment (PDB ID: 9BRX for **11**). Key hydrogen bonds are shown as black dashes.

Class A compounds share a similar binding pocket to other known SARS-CoV-2 PL^Pro^ inhibitors, GRL-0617 and the peptide-derived VIR-250.^26^ Like other inhibitors that bind to the S4 subsite, fragment **7** engages in π–π stacking interactions between their aromatic ring and Y268 (Figure 4C). This induces a conformational change of the BL2 loop from the unbound PL^Pro^ structure to form the exterior wall of the binding site. Fragment **5** binds in a different orientation to most other PL^Pro^ inhibitors, foregoing interaction with Y268 and instead sitting in a small hydrophobic pocket traditionally occupied by the V70 side chain of the ubiquitin-like domain. Additionally, the amine group of **5** forms a hydrogen bond to the side chain of D164, a key binding interaction maintained by GRL-0617 and its analogues. Class B fragments were found to occupy a pocket near the zinc binding site in the PL^Pro^ finger region (Figure 4D). The triazolopyrimidine ring of fragment **11** is placed in a pocket containing V187, Q195, T197, T225, and K232 with a hydrogen bond to a water molecule bridging K232, while its phenyl group is extended into the hydrophobic pocket formed by V188, G193, and C192 next to the zinc site. Interestingly, the chemical structures of the class A fragments (e.g., **6** and **7**) and class B fragment (e.g., **11**) are similar in their primary structures, yet they bind to distinct sites on the protein based on the different NMR shift patterns and the cocrystal structures. Despite their structural similarity, the methyl group at N-1 is the key to the binding preference of the compounds. This nitrogen is observed to create a hydrogen bond to a water molecule, which links to K232 in the finger region. Once it has been methylated, the hydrogen bond formation is disrupted, resulting in binding to the S4 subsite in the palm region.

Although there are well-defined binding pockets and clear avenues for elaboration in both classes of fragment hits, the increased flexibility of the finger region and variability in binding pose make the elaboration of class B fragments a more challenging prospect. Additionally, the distance of the zinc fingers from the active site of PL^Pro^ and the lack of conformational change associated with fragment binding raise concerns as to whether class B compounds can modulate the activity of the protein. Therefore, we developed an enzymatic inhibition assay to assess the inhibitory capabilities of our hit fragments. Full-length PL^Pro^ with intact C111 was incubated with various class A and B fragments with NMR K_d_’s ranging from 250 to 500 μM followed by the addition of a fluorescently labeled substrate (Ac-RLKGG-AMC), and the rate of peptide cleavage was measured by a change in fluorescence. All class A fragments displayed some degree of inhibition of PL^Pro^ at 1 mM with the most active fragment **8** showing 62% inhibition (Figure 5). However, no inhibition was observed for any of the class B fragments, despite many having a higher *K*_d_ than their active class A counterparts, suggesting that ligand binding at the zinc finger region of the protein is not capable of modulating PL^pro^ catalytic activity. Due to the lack of inhibition observed by the class B fragment series, our efforts to develop small molecule inhibitors of PL^Pro^ have been focused on the elaboration of class A molecules.

**Figure 5:**
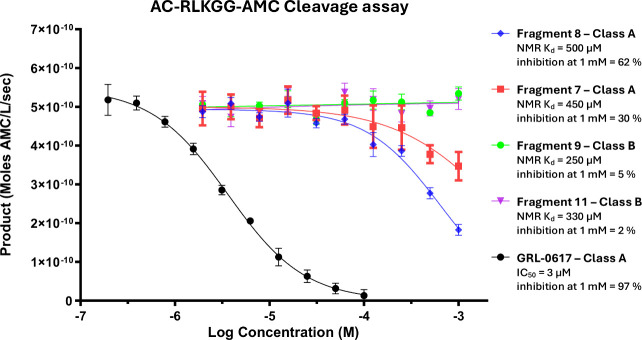
Enzymatic inhibition of previously reported PL^pro^ inhibitor GRL-0617 and initial class A & B fragment hits with their NMR K_d_ displayed.

There is a clear need to develop novel small molecule inhibitors of SARS-CoV-2 to improve patient outcomes and overcome the emergence of drug resistant strains. PL^Pro^ is a critical enzyme for viral replication that has yet to be therapeutically targeted, making it a promising target for a drug discovery campaign. We have conducted a primary fragment screen against PL^Pro^ using protein observed SOFAST-HMQC NMR and identified 77 compounds that bind PL^Pro^ at two distinct regions of the protein, one of which has not been previously identified. Crucially, all fragment series are structurally distinct from GRL-0617 and its analogues (the only other compounds reported to bind PL^Pro^), making them a promising starting point for development of the first novel inhibitors of PL^Pro^. X-ray crystallography was employed to confirm the binding mode of the class A and B fragments revealing well-defined binding pockets at the S4 subsite near the catalytic site and finger subdomain near the zinc site, respectively. While both classes of fragments had clear structure–activity relationship trends and avenues for lead compound expansion, class B fragments were unfortunately not capable of inhibiting the enzymatic reaction of PL^Pro^. Thus, class A fragments are more attractive to elaborate structure-based drug development to create a novel class of small molecule therapeutics to treat SARS-CoV-2 and other coronavirus infections.

## Supporting Information Available

Experimental procedures, X-ray refinement statistics for fragments **5**, **7**, and **11**, and supplementary figures (PDF)

## Accession Codes

Atom coordinates and structure factors for SARS-CoV-2 PL^pro^ complexed with fragments can be accessed in the Protein Data Bank via the following accession codes: 9BRV, fragment 5; 9BRW, fragment 7; 9BRX, fragment 11. The authors will release the atomic coordinates upon article publication.

## Abbreviations

AMC: 7-amino-4-methylcoumarin
BME: β-mercaptoethanol
Covid-19: coronavirus disease 2019
CSP: chemical shift perturbations
IPTG: isopropyl β-d-1-thiogalactopyranoside
ISG-15: the ubiquitin and interferon-stimulated gene 15
NSP: nonstructural protein
PL^pro^: papain-like protease
PMSF: phenylmethylsulfonyl fluoride
SARS-CoV-2: severe acute respiratory syndrome coronavirus 2
SOFAST-HMQC: band-selective optimized flip angle short transient heteronuclear multiple quantum coherence
Ubl: ubiquitin-like

## References

[ref1] Eastman R. T.; Roth J. S.; Brimacombe K. R.; Simeonov A.; Shen M.; Patnaik S.; Hall M. D. Remdesivir: a review of its discovery and development leading to emergency use authorization for treatment of COVID-19. ACS central Science 2020, 6 (5), 672–683. 10.1021/acscentsci.0c00489. 32483554 PMC7202249

[ref2] Imran M.; Kumar Arora M.; Asdaq S. M. B.; Khan S. A.; Alaqel S. I.; Alshammari M. K.; Alshehri M. M.; Alshrari A. S.; Mateq Ali A.; Al-Shammeri A. M.; et al. Discovery, development, and patent trends on molnupiravir: a prospective oral treatment for COVID-19. Molecules 2021, 26 (19), 5795 10.3390/molecules26195795. 34641339 PMC8510125

[ref3] Owen D. R.; Allerton C. M.; Anderson A. S.; Aschenbrenner L.; Avery M.; Berritt S.; Boras B.; Cardin R. D.; Carlo A.; Coffman K. J.; et al. An oral SARS-CoV-2 Mpro inhibitor clinical candidate for the treatment of COVID-19. Science 2021, 374 (6575), 1586–1593. 10.1126/science.abl4784. 34726479

[ref4] Duan Y.; Zhou H.; Liu X.; Iketani S.; Lin M.; Zhang X.; Bian Q.; Wang H.; Sun H.; Hong S. J.; et al. Molecular mechanisms of SARS-CoV-2 resistance to nirmatrelvir. Nature 2023, 622 (7982), 376–382. 10.1038/s41586-023-06609-0. 37696289

[ref5] Hu Y.; Lewandowski E. M.; Tan H.; Zhang X.; Morgan R. T.; Zhang X.; Jacobs L. M.; Butler S. G.; Gongora M. V.; Choy J.; et al. Naturally occurring mutations of SARS-CoV-2 main protease confer drug resistance to nirmatrelvir. ACS Cent. Sci. 2023, 9 (8), 1658–1669. 10.1021/acscentsci.3c00538. 37637734 PMC10451032

[ref6] Iketani S.; Mohri H.; Culbertson B.; Hong S. J.; Duan Y.; Luck M. I.; Annavajhala M. K.; Guo Y.; Sheng Z.; Uhlemann A.-C.; et al. Multiple pathways for SARS-CoV-2 resistance to nirmatrelvir. Nature 2023, 613 (7944), 558–564. 10.1038/s41586-022-05514-2. 36351451 PMC9849135

[ref7] Gordon D. E.; Jang G. M.; Bouhaddou M.; Xu J.; Obernier K.; White K. M.; O’Meara M. J.; Rezelj V. V.; Guo J. Z.; Swaney D. L.; et al. A SARS-CoV-2 protein interaction map reveals targets for drug repurposing. Nature 2020, 583 (7816), 459–468. 10.1038/s41586-020-2286-9. 32353859 PMC7431030

[ref8] Satarker S.; Nampoothiri M. Structural proteins in severe acute respiratory syndrome coronavirus-2. Archives of medical research 2020, 51 (6), 482–491. 10.1016/j.arcmed.2020.05.012. 32493627 PMC7247499

[ref9] V’kovski P.; Kratzel A.; Steiner S.; Stalder H.; Thiel V. Coronavirus biology and replication: implications for SARS-CoV-2. Nature Reviews Microbiology 2021, 19 (3), 155–170. 10.1038/s41579-020-00468-6. 33116300 PMC7592455

[ref10] Mukherjee R.; Dikic I. Proteases of SARS Coronaviruses. Encyclopedia of Cell Biology 2023, 930 10.1016/B978-0-12-821618-7.00111-5.

[ref11] Tan H.; Hu Y.; Jadhav P.; Tan B.; Wang J. Progress and challenges in targeting the SARS-CoV-2 papain-like protease. Journal of medicinal chemistry 2022, 65 (11), 7561–7580. 10.1021/acs.jmedchem.2c00303. 35620927 PMC9159073

[ref12] Shin D.; Mukherjee R.; Grewe D.; Bojkova D.; Baek K.; Bhattacharya A.; Schulz L.; Widera M.; Mehdipour A. R.; Tascher G.; et al. Papain-like protease regulates SARS-CoV-2 viral spread and innate immunity. Nature 2020, 587 (7835), 657–662. 10.1038/s41586-020-2601-5. 32726803 PMC7116779

[ref13] Yuan W.; Krug R. M. Influenza B virus NS1 protein inhibits conjugation of the interferon (IFN)-induced ubiquitin-like ISG15 protein. EMBO J. 2001, 20, 362 10.1093/emboj/20.3.362. 11157743 PMC133459

[ref14] Haas A. L.; Ahrens P.; Bright P.; Ankel H. Interferon induces a 15-kilodalton protein exhibiting marked homology to ubiquitin. J. Biol. Chem. 1987, 262 (23), 11315–11323. 10.1016/S0021-9258(18)60961-5. 2440890

[ref15] Blomstrom D. C.; Fahey D.; Kutny R.; Korant B. D.; Knight E. Jr Molecular characterization of the interferon-induced 15-kDa protein. Molecular cloning and nucleotide and amino acid sequence. J. Biol. Chem. 1986, 261 (19), 8811–8816. 10.1016/S0021-9258(19)84453-8. 3087979

[ref16] Perng Y.-C.; Lenschow D. J. ISG15 in antiviral immunity and beyond. Nature Reviews Microbiology 2018, 16 (7), 423–439. 10.1038/s41579-018-0020-5. 29769653 PMC7097117

[ref17] Lenschow D. J.; Lai C.; Frias-Staheli N.; Giannakopoulos N. V.; Lutz A.; Wolff T.; Osiak A.; Levine B.; Schmidt R. E.; García-Sastre A.; et al. IFN-stimulated gene 15 functions as a critical antiviral molecule against influenza, herpes, and Sindbis viruses. Proc. Natl. Acad. Sci. U. S. A. 2007, 104 (4), 1371–1376. 10.1073/pnas.0607038104. 17227866 PMC1783119

[ref18] Lai C.; Struckhoff J. J.; Schneider J.; Martinez-Sobrido L.; Wolff T.; García-Sastre A.; Zhang D.-E.; Lenschow D. J. Mice lacking the ISG15 E1 enzyme UbE1L demonstrate increased susceptibility to both mouse-adapted and non-mouse-adapted influenza B virus infection. Journal of virology 2009, 83 (2), 1147–1151. 10.1128/JVI.00105-08. 19004958 PMC2612374

[ref19] Wydorski P. M.; Osipiuk J.; Lanham B. T.; Tesar C.; Endres M.; Engle E.; Jedrzejczak R.; Mullapudi V.; Michalska K.; Fidelis K.; et al. Dual domain recognition determines SARS-CoV-2 PLpro selectivity for human ISG15 and K48-linked di-ubiquitin. Nat. Commun. 2023, 14 (1), 2366 10.1038/s41467-023-38031-5. 37185902 PMC10126577

[ref20] Fu Z.; Huang B.; Tang J.; Liu S.; Liu M.; Ye Y.; Liu Z.; Xiong Y.; Zhu W.; Cao D.; et al. The complex structure of GRL0617 and SARS-CoV-2 PLpro reveals a hot spot for antiviral drug discovery. Nat. Commun. 2021, 12 (1), 488 10.1038/s41467-020-20718-8. 33473130 PMC7817691

[ref21] Ratia K.; Pegan S.; Takayama J.; Sleeman K.; Coughlin M.; Baliji S.; Chaudhuri R.; Fu W.; Prabhakar B. S.; Johnson M. E.; et al. A noncovalent class of papain-like protease/deubiquitinase inhibitors blocks SARS virus replication. Proc. Natl. Acad. Sci. U. S. A. 2008, 105 (42), 16119–16124. 10.1073/pnas.0805240105. 18852458 PMC2571001

[ref22] Tan B.; Zhang X.; Ansari A.; Jadhav P.; Tan H.; Li K.; Chopra A.; Ford A.; Chi X.; Ruiz F. X.; et al. Design of a SARS-CoV-2 papain-like protease inhibitor with antiviral efficacy in a mouse model. Science 2024, 383 (6690), 1434–1440. 10.1126/science.adm9724. 38547259 PMC12178660

[ref23] Shen Z.; Ratia K.; Cooper L.; Kong D.; Lee H.; Kwon Y.; Li Y.; Alqarni S.; Huang F.; Dubrovskyi O.; et al. Design of SARS-CoV-2 PLpro inhibitors for COVID-19 antiviral therapy leveraging binding cooperativity. J. Med. Chem. 2022, 65 (4), 2940–2955. 10.1021/acs.jmedchem.1c01307. 34665619 PMC8547495

[ref24] Garnsey M.; Robinson M.; Nguyen L.; Cardin R.; Tillotson J.; Mashalidis E.; Aijia Y.; Aschenbrenner L.; Balesano A.; Behzadi A.; et al. Discovery of orally active SARS-CoV-2 papain-like protease (PLpro) inhibitors. bioRxiv 2024, 10.1101/2024.01.26.577395. PMC11364104 39213347

[ref25] Sanders B. C.; Pokhrel S.; Labbe A. D.; Mathews I. I.; Cooper C. J.; Davidson R. B.; Phillips G.; Weiss K. L.; Zhang Q.; O’Neill H.; et al. Potent and selective covalent inhibition of the papain-like protease from SARS-CoV-2. Nat. Commun. 2023, 14 (1), 1733 10.1038/s41467-023-37254-w. 36977673 PMC10044120

[ref26] Rut W.; Lv Z.; Zmudzinski M.; Patchett S.; Nayak D.; Snipas S. J.; El Oualid F.; Huang T. T.; Bekes M.; Drag M.; Olsen S. K. Activity profiling and crystal structures of inhibitor-bound SARS-CoV-2 papain-like protease: A framework for anti-COVID-19 drug design. Sci. Adv. 2020, 6 (42), eabd4596 10.1126/sciadv.abd4596. 33067239 PMC7567588

[ref27] Schanda P.; Kupče E̅.; Brutscher B. SOFAST-HMQC experiments for recording two-dimensional deteronuclear correlation spectra of proteins within a few seconds. Journal of biomolecular NMR 2005, 33, 199–211. 10.1007/s10858-005-4425-x. 16341750

[ref28] Hajduk P. J.; Huth J. R.; Fesik S. W. Druggability indices for protein targets derived from NMR-based screening data. Journal of medicinal chemistry 2005, 48 (7), 2518–2525. 10.1021/jm049131r. 15801841

[ref29] Shiraishi Y.; Shimada I. NMR Characterization of the Papain-like Protease from SARS-CoV-2 Identifies the Conformational Heterogeneity in Its Inhibitor-Binding Site. J. Am. Chem. Soc. 2023, 145 (30), 16669–16677. 10.1021/jacs.3c04115. 37478405

