# Supplementary material for: Fragment-Based Screen of SARS-CoV-2 Papain-like Protease (PLpro)
Source: ACS Med Chem Lett. 2024 Jul 23;15(8):1351–7. doi: 10.1021/acsmedchemlett.4c00238 (PMC11318101; doi:10.1021/acsmedchemlett.4c00238)
Supplement: Supplementary file 1 — ml4c00238_si_001.pdf [file ml4c00238_si_001.pdf]

Supplementary Information

Fragment-based screen of SARS-CoV-2 papain-like protease (PL^{pro})

Ashley J. Taylor*[1], Kangsa Amporndanai*[1], Tyson A. Rietz[1], Bin Zhao[1], Anusha Thiruvaipati[1], Qiangqiang Wei[1], Taylor M. South[1], Mackenzie M. Crow[1], Chideraa Apakama[1], John L. Sensintaffar[1], Jason Phan[1], Taekyu Lee[1], Stephen W. Fesik [1,2,3]†.

[1] Department of Biochemistry, Vanderbilt University School of Medicine, Nashville, Tennessee, 37232-0146, United States.

[2] Department of Pharmacology, Vanderbilt University School of Medicine, Nashville, Tennessee, 37232-6600, United States.

[3] Department of Chemistry, Vanderbilt University, Nashville, Tennessee, 37235, United States.

* Co-first authors
†Corresponding author

Corresponding Author Contact

Stephen W. Fesik
Phone: +1 (615) 322-6303; Fax: +1 (615) 875-3236;
Email: Stephen.fesik@vanderbilt.edu

Table of Contents

Experimental Procedures	S2
Protein Expression and Purification	S2
NMR Experiments	S2
Protein Crystallization and Structure Determination	S3
Enzymatic Assay of SARS-CoV-2 PL^{pro}	S3
X-ray data collection and refinement statistics	S4
Supplementary figures	S5

Experimental Procedures

All experiments conducted in this work do not have unexpected or unusually high safety hazards.

Protein Expression and Purification.

The gene containing the ubiquitin-like domain and the catalytic core of SARS-CoV-2 PL^{Pro} (residues 1-315) was synthesized with codon optimization for *Escherichia Coli* and cloned in the pET28a(+) vector by GenScript. We made PL^{Pro} constructs to optimize the protein for NMR-based fragment screen (residues 71-314 with C111S and C270S), X-ray crystallography (residues 1-314 with C111S and C270S) and the enzymatic assays (residues 1-315 with C270S) were obtained by site mutagenesis. All PL^{Pro} plasmids were transformed into the BL21(DE3) strain *E. Coli*. The bacteria were cultured in Luria-Bertani broth or M9 minimal media containing $^{15}NH_4Cl$ supplemented with 50mg/mL Kanamycin at 37°C until the optimal density at 600nm reached 0.8 before inducing protein expression by the addition of 0.1mM IPTG and 0.1mM $ZnCl_2$ at 18°C for 20 hours. The cell pellet was harvested by centrifugation at 5,000g for 15 minutes, re-suspended in lysis buffer (50mM Tris pH 7.0, 500mM NaCl, 5%glycerol, 10mM imidazole, 5mM BME, 0.1% Triton X-100 and 1mM PMSF), and lysed in APV2000 lab homogenizer (SPX flow). Cell lysate was centrifuged at 15,000g for 45 minutes and loaded onto HisTrap FF column (Cytiva). The column was washed with 10 column volumes of Buffer A (50mM Tris pH 7.0, 500mM NaCl, 5%glycerol, 10mM imidazole, 5mM BME) and eluted with Buffer B (50mM Tris pH 7.0, 500mM NaCl, 5%glycerol, 500mM imidazole, 5mM BME) using a linear gradient program from 0 to 100% Buffer B over 10 column volumes. To the fractions containing PL^{Pro}, Thrombin was added to remove the 6xHis tag and dialyzed against Buffer A without imidazole overnight. Then, Tag-cleaved PL^{Pro} was loaded on a HisTrap column. The flowthrough was concentrated and subjected to HiLoad 26/600 Superdex75 pg (Cytiva) and eluted using Buffer C (20mM HEPES pH 7.0, 150mM NaCl, 3mM DTT) for the NMR-based fragment screen or Buffer D (25mM Tris pH 7.0, 150mM NaCl, 3mM DTT) for X-ray crystallography and the enzymatic assays. Protein concentration was quantified by the Pierce 660nm assay (ThermoFisher).

NMR Experiments.

All NMR experiments were performed at 25°C using a 600 MHz Bruker Avance III spectrometer equipped with a 5mm single-axis x-gradient cryoprobe and a Bruker SampleJet. Gradient-enhanced, two-dimensional ^1H-^{15}N heteronuclear multiple-quantum coherence (SOFAST-HMQC) spectra of PL^{Pro} were recorded using 24 scans of 12-minute acquisition times and analyzed using Topspin 4.1.4 (Bruker). Our in-house fragment library of 13,824 compounds was screened as mixtures of 12 fragments prepared in twelve 96-well plates. Each NMR sample was made of 15μM of ^{15}N-labeled PL^{Pro}, 800μM of each fragment, and 5% DMSO-d_6 for spectrometer locking in 5mm-diameter NMR tubes. Hit mixtures were identified by comparing the chemical shifts of the backbone resonances to a ligand-free PL^{Pro} spectrum and then deconvoluted by screening individual fragments.

SOFAST-HMQC titration experiments were used to determine binding affinity of the fragment hits identified from the screen. The changes in ^1H-^{15}N chemical shifts of backbone resonances upon the addition of increasing concentrations of the fragments (0.0625-2mM) were analyzed. The binding affinities (K_ds) of the fragments were calculated using the Hill's equation model in Prism 10 (GraphPad).

Protein Crystallization and Structure Determination.
7mg/mL PL^{Pro} was mixed with a 200mM DMSO stock of the desired fragments at the final concentrations of 5mM fragment and 2.5%DMSO and incubated at 4°C overnight. Crystallization drops of 0.5µL of PL^{Pro} -fragment + 0.5µL of reservoir solution were set as sitting drops and incubated at 18°C allowing vapor diffusion against the corresponding reservoir solution. The crystals were obtained in 0.2M magnesium formate, 15-25%PEG3350 (Fragments **5** and **11**) or 0.2M sodium citrate, 15%-25%PEG3350 (Fragment **7**) and cryo-protected in mother liquor supplemented with 20% ethylene glycol before being flash-frozen in liquid nitrogen. X-ray experiments were performed at 100K on the Life Sciences Collaborative Access Team (LS-CAT) Sector-21 beamlines at the Advanced Photon Source (APS), Argonne National Laboratory. Diffraction data were indexed, integrated, and scaled with HKL2000. Phasing was accomplished by molecular replacement with Phaser using the structure of SARS-CoV-2 PL^{pro} with C111S (PDB:6WRH) as starting model. Ligand models were built by AceDRG and manually added to the corresponding electron density. PL^{Pro} -ligand co-crystal structures were determined by several cycles of refinement using Phenix and manual modelling with COOT.

Enzymatic Assay of SARS-CoV-2 PL^{pro}
All compounds were stored in 200 mM stock in DMSO. Dose responses of the compounds were generated using an ECHO 555 Liquid Handler (Labcyte, Inc.) in a 384 well black polystyrene flat bottom plate with a non-binding surface (Corning p/n 3575). 10 µL of recombinant PL^{Pro} was added at a concentration of 160 nM and incubated for 30 minutes, followed by the addition of 10 µL of Ac-RLKGG-AMC at a concentration of 60 µM (The final concentrations of enzyme and substrate are 80 nM and 30 µM, respectively, with a DMSO concentration of 5%). AMC product formation was measured at 5 minutes and 15 minutes, and the enzyme activity was expressed as moles AMC $L^{-1}sec^{-1}$. GRL-0617 served as a PL^{Pro} positive control inhibitor (Selleckchem). Positive control wells contained enzyme, substrate, and vehicle, while negative control wells had substrate and vehicle minus the enzyme. The dose range for the test compounds was 2.0µM - 1mM.

X-ray data collection and refinement statistics

Table S1. X-ray data collection and refinement statistics for fragments bind to PL^{pro}.

Compound<br>PDB Accession code	Fragment **5**<br>9BRV	Fragment **7**<br>9BRW	Fragment **11**<br>9BRX
**Data Collection**			
Space Group	P 32	P21 21 21	P 32
Cell Dimensions			
a, b, c (Å)	83.281, 83.281,132.969	98.82, 119.86, 125.39	81.998, 81.998, 134.674
α, β, γ (°)	90.00, 90.00, 120.00	90.00, 90.00, 90.00	90.00, 90.00, 120.00
Resolution (Å)	22.61-2.48<br>(2.58-2.48)	29.96-2.50<br>(2.56-2.50)	27.85-1.80<br>(1.83-1.80)
R_{merge} (%)	0.206 (0.752)	0.166 (0.550)	0.206 (0.682)
Mean I / σI	10.6 (2.1)	11.0 (2.2)	9.0 (3.2)
Completeness (%)	99.8 (100)	99.7 (89.3)	99.5 (95.4)
Redundancy	17.1 (16.6)	8.2 (6.8)	9.4 (9.6)
**Structure Refinement**			
No. Reflections	63,093	52,000	49,278
R_{work}/R_{free}	0.1949/0.2263	0.2015/0.2443	0.1728/0.1898
R.m.s. deviations			
Bond lengths (%)	0.008	0.005	0.007
Bond angles (%)	1.384	0.861	0.872
Ramachandran			
Preferred regions (%)	96.04	95.01	97.13
Allowed regions (%)	3.96	4.83	2.87
Disallowed regions (%)	0	0.16	0

*High resolution shells are in parentheses.

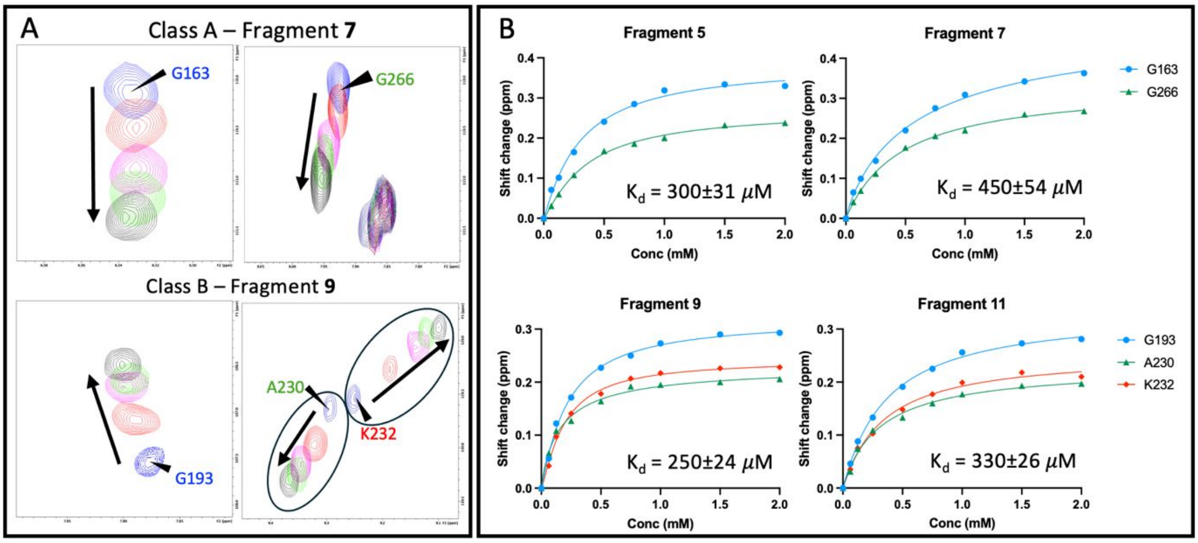

Figure S1. Chemical shift perturbations and affinity measurement for fragments **5** and **9**. (A) Sections of the ^{1}H-^{15}N SOFAST-HMQC spectrum, with the overlaid of the ligand-free PL^{pro} spectrum (blue) and PL^{pro} spectra with increasing fragment concentrations (0.125mM red, 0.75mM pink, 1mM green and 2mM black) (B) Titration curves of selected fragments. Reported K$_d$s were average K$_d$s from peaks.

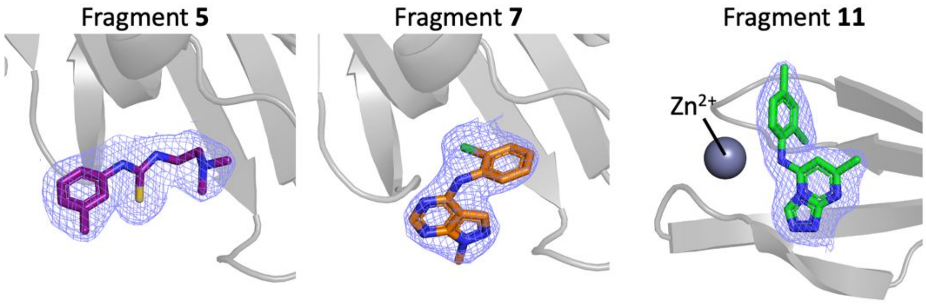

Figure S2. Electron density maps of fragments **5**, **7** and **11** contoured at 1σ level in PL^{pro} crystal structures.
